# Degradation and Characterisation of Electrospun Polycaprolactone (PCL) and Poly(lactic-co-glycolic acid) (PLGA) Scaffolds for Vascular Tissue Engineering

**DOI:** 10.3390/ma14174773

**Published:** 2021-08-24

**Authors:** Morteza Bazgir, Wei Zhang, Ximu Zhang, Jacobo Elies, Morvarid Saeinasab, Phil Coates, Mansour Youseffi, Farshid Sefat

**Affiliations:** 1 Department of Biomedical and Electronics Engineering, School of Engineering, University of Bradford, Bradford BD7 1DP, UK; m.bazgir@student.bradford.ac.uk (M.B.); m.youseffi@bradford.ac.uk (M.Y.); 2 State Key Laboratory of Polymer Materials Engineering, Polymer Research Institute, Sichuan University, Chengdu 610065, China; weizhang@scu.edu.cn; 3 Advanced Polymer Materials Research Center, Sichuan University, Shishi 362700, China; 4 Chongqing Key Laboratory of Oral Disease and Biomedical Sciences and Chongqing Municipal Key Laboratory of Oral Biomedical Engineering of Higher Education, Stomatological Hospital of Chongqing Medical University, Chongqing 401174, China; zhangximu@hospital.cqmu.edu.cn; 5 Faculty of Life Sciences, School of Pharmacy and Medical Sciences, University of Bradford, Bradford BD7 1DP, UK; j.eliesgomez@bradford.ac.uk; 6 Department of Biology, Faculty of Science, Ferdowsi University of Mashhad, Mashhad 9177948974, Iran; m.saeinasab@gmail.com; 7 Interdisciplinary Research Centre in Polymer Science and Technology (Polymer IRC), University of Bradford, Bradford BD7 1DP, UK; P.D.Coates@bradford.ac.uk

**Keywords:** electrospinning, polycaprolactone (PCL), Poly(lactic-co-glycolic acid) (PLGA), tissue engineering, porous biodegradable membrane, degradation, tensile test

## Abstract

The current study aimed to evaluate the characteristics and the effects of degradation on the structural properties of Poly(lactic-co-glycolic acid) (PLGA)- and polycaprolactone (PCL)-based nanofibrous scaffolds. Six scaffolds were prepared by electrospinning, three with PCL 15% (*w*/*v*) and three with PLGA 10% (*w*/*v*), with electrospinning processing times of 30, 60 and 90 min. Both types of scaffolds displayed more robust mechanical properties with increased spinning times. The tensile strength of both scaffolds with 90-min electrospun membranes did not show a significant difference in their strengths, as the PCL and PLGA scaffolds measured at 1.492 MPa ± 0.378 SD and 1.764 MPa ± 0.7982 SD, respectively. All membranes were shown to be hydrophobic under a wettability test. A degradation behaviour study was performed by immersing all scaffolds in phosphate-buffered saline (PBS) solution at room temperature for 12 weeks and for 4 weeks at 37 °C. The effects of degradation were monitored by taking each sample out of the PBS solution every week, and the structural changes were investigated under a scanning electron microscope (SEM). The PCL and PLGA scaffolds showed excellent fibre structure with adequate degradation, and the fibre diameter, measured over time, showed slight increase in size. Therefore, as an example of fibre water intake and progressive degradation, the scaffold’s percentage weight loss increased each week, further supporting the porous membrane’s degradability. The pore size and the porosity percentage of all scaffolds decreased substantially over the degradation period. The conclusion drawn from this experiment is that PCL and PLGA hold great promise for tissue engineering and regenerative medicine applications.

## 1. Introduction

Tissue engineering research holds promise for treating tissue loss and severe organ injuries/failure because human tissue is a diverse and complex system that requires various strategies in different locations [1]. Although allografts and autografts remain the clinical gold standard techniques for treating most organ failures, they are not guaranteed successful methods for treating such defects, as allografts can be rejected by the recipient’s body and cause inflammation or necrosis if not appropriately treated before and after implementation [2,3,4]. However, when comparing these two treatment methods, in most cases, allografts are considered to be more advantageous, as they prevent donor site pain and morbidity, which are often caused by autograft harvesting [5,6]. While the allograft is a standard method used for treating patients with faulty organ/tissues, mostly, this type of transplantation requires the patient to be on the waiting list to receive a matching organ/tissue; it also raises many ethical issues including how the organ has been obtained, and some recipients’ religious beliefs do not permit them to receive such treatments. Therefore, it is desirable to develop a potential biodegradable synthetic membrane that can facilitate, regenerate, and replace damaged human tissues or organs without the need for secondary revision surgery.

Biomaterials play a crucial role in the field of tissue engineering. Recently, many kinds of research have been conducted to measure the feasibility of using scaffolds made from biomaterials for tissue regeneration purposes, especially biodegradable polymeric scaffolds [7,8,9,10,11]. Some of these kinds of biodegradable polymers have shown that three-dimensional scaffolds can allow the diffusion of nutrients and also support cell adhesion, proliferation and differentiation for functional tissue regeneration [12,13,14]. More precisely, a good amount of research has been conducted by different researchers globally using various polymeric and synthetic biomaterials for many applications within the human body which mainly have used electrospinning technique including in the breast [15], bone [16,17], nerves [18], dental [19,20], skin [21,22,23], cornea and contact lenses [24,25,26,27,28,29,30], blood vessels [31], ligaments [32], diaphragm [33], trachea [34,35], lung [36], cartilage [37], bladder [38] and intestine [39], and all of the mentioned tissues have involved the same principle.

An ideal tissue-engineered scaffold depends on the location of its intended use, and it should have many specific characteristics, such as fabricated polymeric scaffolds should be biodegradable, biocompatible, have appropriate mechanical properties, be porous with an ideal pore size for allowing cells and nutrition to migrate within in the scaffold structure and mimic the native extracellular matrix (ECM) [40,41,42]. Pore size usually measures the gap between fibrous structures using various techniques, which are very crucial. In this study, pore size was measured using ImageJ software. Therefore, the choice of biomaterials and the kind of scaffold fabrication technique have significant roles in determining the tissue-engineered membrane’s required characteristics and its success. Scaffolds from synthetic Poly(ε-caprolactone) and Poly(lactide-co-glycolide) polymers have been extensively studied for various applications including skin and vascular grafts and neural, cartilage, and bone tissue engineering [1,43,44,45,46,47,48]. Many manufacturing methods can be used to fabricate 3D scaffolds from these biodegradable synthetic polymers. However, in recent years, there has been more attention drawn toward the usability of the electrospinning process in the field of tissue engineering, as it can produce a three-dimensional, nano-scale fibrous membrane with extremely high surface and structural porosity [49,50]. A variety of components combined with the ability to precisely control mechanical properties, structural properties and work capacity have led to the widespread use of electrospinning technology in the regenerative medicine field [51]. 

The pore size and overall porosity of the electrospun scaffolds mainly depend on the polymeric fibre distribution as well as the diameter of the fabricated fibres [52]. In most scaffolds tissue-engineered via an electrospinning technique, studies have seen a similar trend: The wider the fibre diameter, the wider pore size will be [53,54]. However, there is a significant drawback to increasing pore size and the scaffold’s overall porosity; this will reduce the mechanical stability of the scaffold [55,56,57]. Therefore, it is mandatory to have the optimum porosity and mechanical strength for engineered scaffolds. The development of electrospun membranes with random and aligned fibres mimics the natural ECM, and it has generated significant interest in various tissue engineering applications [58,59]. Due to the impact that the electrospinning process has on scaffold morphology, mechanical properties and biodegradability, it has been selected as the primary method for producing synthetic polymeric scaffolds. The current study aimed to evaluate the structural morphology, wettability, mechanical properties as well as the effects of degradation on electrospun PCL and PLGA structures at room temperature and 37 °C. Both PCL and PLGA were selected in this study of a vascular tissue engineering application due to their great biocompatibility, degradation rate and many other factors, which made these two biopolymers very suitable for the fabrication of artificial blood vessels. In this study, all parameters were tested on both tubular and flat membrane scaffolds. The tubular scaffold was made and tested mainly due to its application in the field of vascular tissue engineering. 

## 2. Materials and Methods

### 2.1. Materials

PURASORB Poly(lactic-co-glycolic acid) 82:18 was obtained from Corbion, Amsterdam, Netherlands and Poly(ε-caprolactone) with an average molecular weight of Mn 80,000 and density of 1.145 g/mL at room temperature was purchased from Sigma-Aldric, St. Louis, Missouri, USA). N,N-dimethylformamide (DMF), tetrahydrofuran (THF) and chloroform (CF), supplied by Fisher Scientific, Loughborough, UK and without prior purification, were used as solvents.

### 2.2. Solution Preparation and Electrospinning Procedure 

The polymeric solutions were prepared by dissolving 4.5 g of PCL pellets in 25.5 g of chloroform and 3 g of PLGA in 13.5 g of THF and 13.5 g of DMF (50:50). The solutions were placed on a magnetic stirrer in a sealed glass container for a minimum of 16 h; next, when the polymer pellets were entirely dissolved in the solution, the glass vials were placed in the ultrasonic bath for an additional 2 h to eliminate any bubbles that had been produced during the mixing procedure.

**Figure 1:**
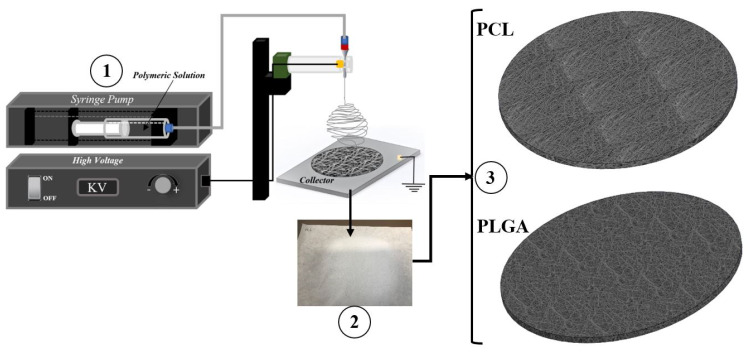
Schematics of the production of flat sheet electrospun scaffolds (**1**) Syringe pump and power source, (**2**) collector, (**3**) electrospun sheet

The basic electrospinning setup is schematically shown in Figure 1. When the polymeric solutions were ready for the electrospinning procedure, using a 16 G needle, the solution was drawn in a sterile NORM-JECT 20 mL syringe and mounted to the syringe pump, and then a tube with an internal capillary delivery of 1 mm diameter was attached. For this study, the 20 G needle was used. Three different electrospun meshes were produced using three different time intervals (30 min, 60 min and 90 min). We adjusted the high voltage each time according to the behaviour of the solution at the needle tip. The voltage was increased each time until a Tylor cone was observed. Table 1 below provides a summary of the parameters recorded during the electrospinning procedure. After each electrospinning procedure, the fabricated scaffolds were then placed in the vacuum chamber at room temperature for a minimum of 24 h to remove any remaining solvent residuals.

### 2.3. Wettability Test 

The wettability of electrospun PCL and PLGA nanofibrous scaffolds were calculated by static contact angle instrument (VCA-Optima, AST, Inc., Billerica, MA, USA). Glass slides were used to hold the scaffolds flat for analysis. A micro-syringe was used to drop 3 µL of deionised water onto the surface of the membrane. Five seconds later, an image was captured, and the contact angles of the droplet were analysed and calculated. Generally, a contact angle of 90° or less indicates better wettability, with a hydrophilic surface, while a contact angle greater than 90° signifies that the surface of the measured materials is hydrophobic.

### 2.4. Scaffold Morphology Characterisation 

To obtain a surface morphological analysis of each sample, a 5 mm^2^ square was cut from the dried, electrospun PCL and PLGA scaffolds before and during the degradation test. This process was repeated every week to evaluate the morphological changes to the membrane during the degradation process. Samples were then viewed using a field emission scanning electron microscope (Hitachi TM3000, Tokyo, Japan) at 1200× magnification and an acceleration of 5 kV. The average fibre diameter (µm), average pore size (µm^2^) and scaffold surface porosity percentage were determined using SEM-assisted image analysis software. All measurements were performed using ImageJ software (Version: 1.53k14, University of Wisconsin, Madison, WI, USA); this software used a grey level on the SEM image to characterise the micrograph at the original magnification. At least 20 fibres and 20 pores were analysed from each captured image, and the average value was determined for each sample.

### 2.5. Degradation Procedure 

Bioresorbable polymers are designed to degrade within a living body after performing their role. When a researcher or inventor wants to validate their product’s success rate, the most accurate results can be obtained from in vivo testing, either by implanting the tissue-engineered scaffold into an animal or human being. Nevertheless, these types of tests are very hazardous, time-consuming and expensive. However, some non-invasive procedures can be performed within the laboratory environment that can provide the researcher with some preliminary results on how their invented bioresorbable polymeric scaffolds could react if they were implanted within the body. These types of tests are very beneficial within the biomedical and tissue engineering field. In this study, two hydrolytic degradation tests were carried out in a PBS solution. The phosphate-buffered saline solution (PBS) was produced by dissolving five tablets, supplied by Fisher Scientific, Waltham, MA, USA, in 1 L of deionised water (0.1 M, pH 7.4). The considered experiments involved 12 weeks of degradation at room temperature and four weeks at a controlled temperature of 37 °C. All samples were cut into a rectangular shape of approximately 5 mm × 10 mm and then submerged in PBS solution with 0.05% sodium azide (NaN_3_) to prevent microbe growth. The samples were removed from the solution every week and rinsed two times with distilled water to remove any minerals deposited by the PBS solution. These samples were left at room temperature in a sterilised laboratory hood overnight to evaporate any remaining liquid. When the scaffolds were dried, they were weighed and compared with the initial start date. Later, samples were further analysed under SEM to understand the scaffolds’ structural behaviour changes under degradation process conditions.

### 2.6. Tensile Testing Procedure 

The mechanical properties of the electrospun nanofibrous scaffolds were measured with a uniaxial testing machine (MACH-1 mechanical tester) using a single-axis 10 kg load cell under a velocity of 0.5 mm/s at room temperature conditions. All samples (*n* = 3) were prepared in a rectangular shape with dimensions of 35 mm × 6 mm using surgical scissors. The thickness of each sample was measured by both digital micrometer and digital calliper. At least three samples were tested for each type of electrospun mesh.

### 2.7. Statistical Analysis 

All the data (at least triplicate) in this study are expressed as mean ± standard deviations (SD). One-way ANOVA analysis determined statistical differences, and differences were considered statistically significant at *p* < 0.05.

## 3. Results 

### 3.1. Water Contact Angle 

**Figure 2:**
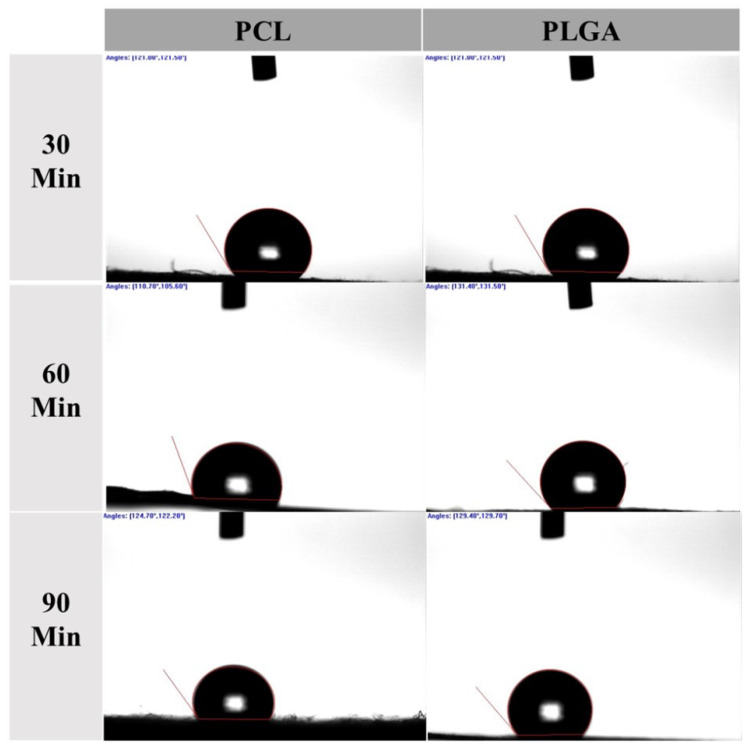
Contact angle measurements using a goniometer to investigate the wettability of both PCL and PLGA scaffolds.

One of the critical factors that can indicate how a scaffold will perform with cells is the surface wettability test [60]. Wettability refers to the hydrophobicity or hydrophilicity of the material. Wettability is generally associated with free surface energy, which is understood as a measure of adhesion energy [61]. A wettability study is essential to determine the scaffold’s solid surface tension, and it also provides information about the strength of the solid/liquid interaction [62]. The smaller the contact angle, the more vital interaction is. Material with a contact angle greater than 90° corresponds to a low wettability, and therefore, it is hydrophobic, and material with a contact angle lower than 90° corresponds to high wettability, and it is hydrophilic. The nature of the hydrophobicity/hydrophilicity of the membrane layer directly influences its use. For example, hydrophobic membranes are preferred when a membrane is required that prevents liquid absorption. However, a scaffold designed for tissue engineering purposes is desired to have some hydrophilic nature to encourage the required cells to penetrate the scaffold pores and proliferate. Overall, due to the use of hydrophobic polymers with high molecular weights, all the samples were found to be hydrophobic, as the contact angles were greater than 90°, which could be problematic for cell attachment and cell proliferation. Surprisingly, the PLGA membranes showed to be slightly more hydrophobic than the PCL membranes. Table 2 below provides the calculated averages of the surface contact angle measurements of all electrospun samples (See Figure 2). 

### 3.2. Degradation Rate 

**Figure 3:**
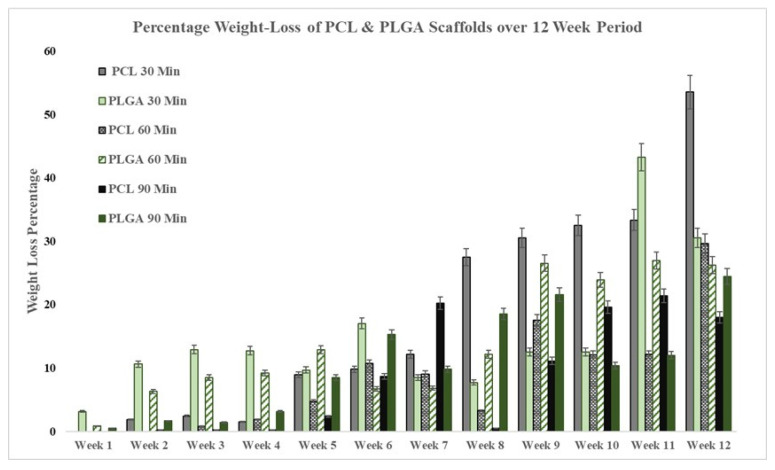
Degradation and weight loss percentage change of PCL and PLGA scaffolds over a 12-week period.

Figure 3 below shows the weight loss percentage (%) for 30, 60 and 90 min electrospun scaffolds for the two driving polymeric solutions, i.e., PCL and PLGA. Temporary scaffolds needed to have a consistent degradation rate for tissue growth, as each of these scaffolds showed different degradation characteristics over the 12 weeks of the degradation period. The mass loss and the changes in the morphology were investigated. Every week, one sample from each scaffold type was removed from the batch and was then dried and lined with an absorbent paper towel to remove any moisture; next, the sample was left in a dry environment for a minimum of 24 h before weight measurement. Due to the scale’s sensitivity, it was difficult to obtain one accurate measurement; therefore, each sample was removed and placed three times before an average was calculated.

According to previous studies by various researchers, the semi-crystallinity of polymers tends to become more crystalline during the degradation period once they are subjected to room temperature; therefore, this matter affects the degradation rate because room temperature is unstable, and scaffolds undergo secondary amorphous phase crystallisation [63,64,65]. The 30- and 60-min PCL scaffolds showed a higher weight loss percentage over 12 weeks compared to the scaffolds’ initial start date weights, but it appeared that the PCL scaffolds electrospun for 90 min degraded slower compared to the 90-min electrospun PLGA scaffolds. These results were expected due to the high ratio of lactic acid in PLGA polymers, making them more hydrophobic and less susceptible to degradation in PBS solution at room temperature [66]. 

### 3.3. Scaffold Morphology 

**Figure 4:**
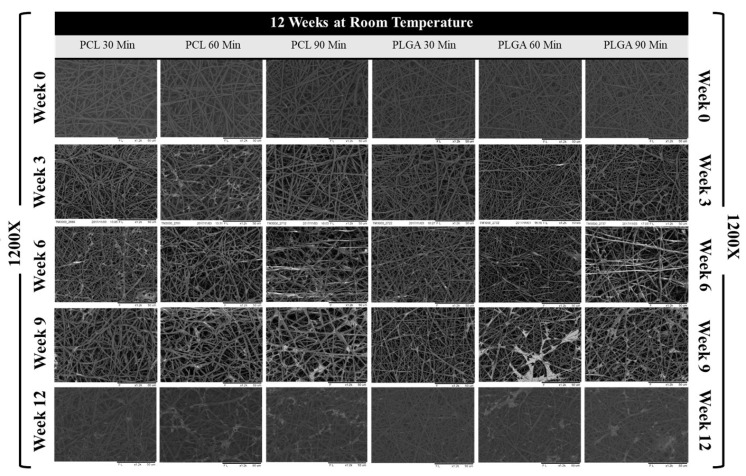
SEM images of electrospun PCL and PLGA scaffolds from weeks 0, 3, 6, 9 and 12 (the degradation period) at 1200× magnification; scale bar: 50 µm.

**Figure 5:**
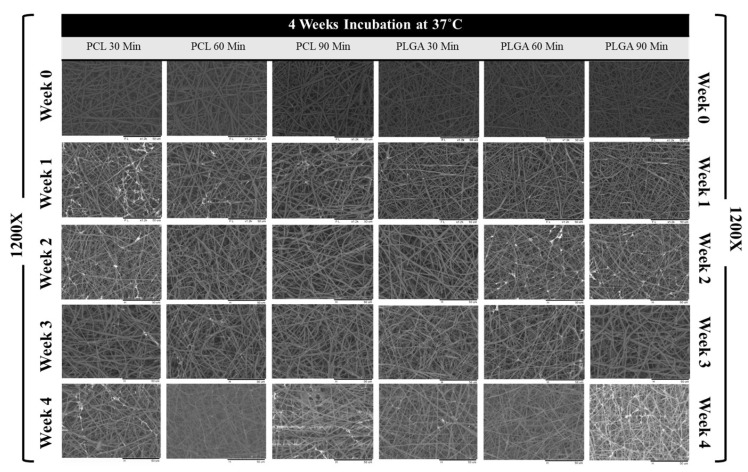
SEM images of electrospun PCL and PLGA scaffolds from weeks 0, 1, 2, 3 and 4 (the degradation period) at 1200× magnification; scale bar: 50 µm.

The degradation process in phosphate-buffered saline solution can impact many aspects of polymeric scaffolds. The saline solution is alkaline, and it can provide helpful information about how these biodegradable scaffolds could perform in vivo in terms of their morphology, fibre diameter, pore size and percentage of surface porosity (%) changes. Figure 4 and Figure 5 below show representative SEM images of all electrospun scaffolds during 12 weeks at room temperature and 4 weeks at a controlled temperature of 37 °C, respectively. These SEM images show that the electrospun membranes formed a very highly interconnected web with adequate surface porosity that was relatively smooth. However, the polymeric solutions’ electrospinning process was optimized, and no jet instabilities and beads were detected. With ImageJ software’s help, further image analysis was carried out, and the average fibre diameter, average pore size and % porosity were measured from these SEM images.

**Figure 6:**
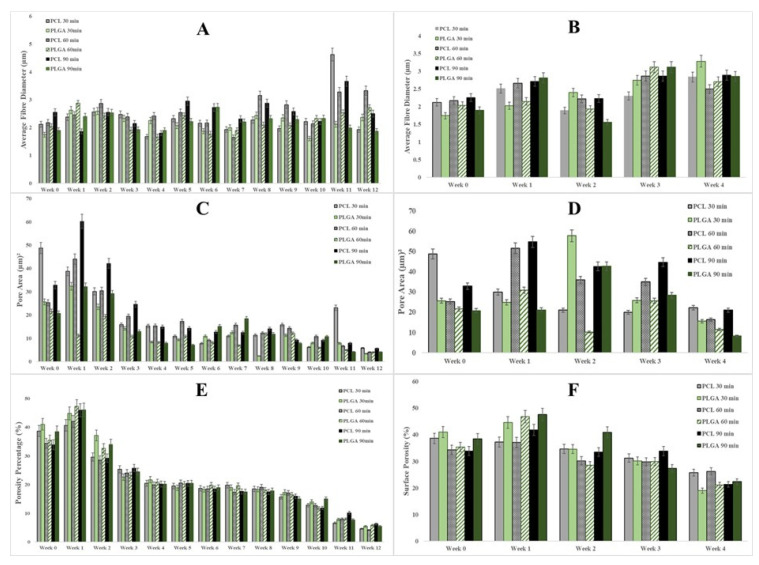
(**A**) Average fibre diameter change (µm) of electrospun scaffolds over a degradation period of 12 weeks at room temperature; (**B**) Average fibre diameter change (µm) of electrospun PCL and PLGA scaffolds over a degradation period of 4 weeks and under temperature-controlled conditions (37 °C); (**C**) Changes in surface pore size (µm^2^) of electrospun PCL and PLGA scaffolds over a 12-week degradation period at room temperature; (**D**) Changes in surface pore size (µm^2^) of electrospun PCL and PLGA scaffolds over a 4-week degradation period under temperature-controlled conditions (37 °C); (**E**) Changes in surface porosity percentages of electrospun PCL and PLGA scaffolds over a degradation period of 12 weeks at room temperature; (**F**) Changes in surface porosity percentages of electrospun PCL and PLGA scaffolds over a degradation period of 4 weeks under temperature-controlled conditions (37 °C).

One of the essential characteristics of an electrospun tissue engineering scaffold is its fibre morphology. The way that fibres connect and accumulate upon each other does not only affect the scaffold’s structural integrity and mechanical properties; it can significantly impact how the cell integrates and proliferates because an increase in the fibre diameter size reduces the overall porosity and pore area volume. The data presented in Figure 6A,B show bar charts of average fibre diameter changes over 12 weeks at room temperature and 4 weeks under controlled conditions at 37 °C, respectively. Because of the moisture absorption of polymer fibres, an increase in the fibre diameter was observed in most scaffolds except for 30- and 90-min PCL scaffolds, in which the fibre diameter reduced by 9.04 (%) and 1.72 (%), respectively. In addition, the fibre diameter of the 90-min PLGA scaffold decreased by 1.38 percent over 12 weeks of degradation at room temperature. However, all of the electrospun scaffolds showed significant increases in their fibre diameters when subjected to a controlled temperature of 37 °C. Remarkably, the 30-min PLGA scaffold had a staggering 88.07% increase in its fibre diameter, as shown in Figure 6A,B below.

On the other hand, although the pore size, or pore volume distribution, between the fibres is of obvious importance, it is somewhat more challenging to characterise and is reported less frequently. Thus far, a couple of methods have been reported to determine the pore size of electrospun interconnected fibrous membranes such as using a liquid extrusion porosimeter/mercury intrusion porosimeter or using ImageJ software [67,68,69,70]. In this case, ImageJ was used to measure pore size area changes on the scaffold surface. Overall, all scaffolds showed a similar trend in their pore size volume, in which the average pore sizes reduced substantially due to scaffolds that were shrunken during both types of degradation processes. Table 3 below provides an accurate calculation of percentage change in pore size for both PCL and PLGA scaffolds. Noticeably, the pore size of some of the scaffolds increased in volume during the first two weeks but then started to decrease in size, but with further analysis, it was concluded that this phenomenon occurred due to the decrease in fibre diameter of these electrospun scaffolds, as it could be noticed in previous figures and tables.

A scaffold that is porous on the surface has several advantages such as increasing surface area and providing more binding sites for drug loading, which are valuable properties for tissue engineering applications. It also helps increase cell attachment and tissue compatibility, and the porous structure influences the roughness and wettability of the frame and the specific permeability process [71]. Figure 6E,F show a bar chart of surface porosity percentage taken each week over 12 weeks at room temperature and 4 weeks at constant 37 °C in an incubator. The total percentage changes in surface porosity of the porous PCL and PLGA membranes throughout the degradation period are listed in Table 3C below, and it shows the same trend as overall pore size change with a significant reduction in its overall pore distribution.

### 3.4. Mechanical Properties 

**Figure 7:**
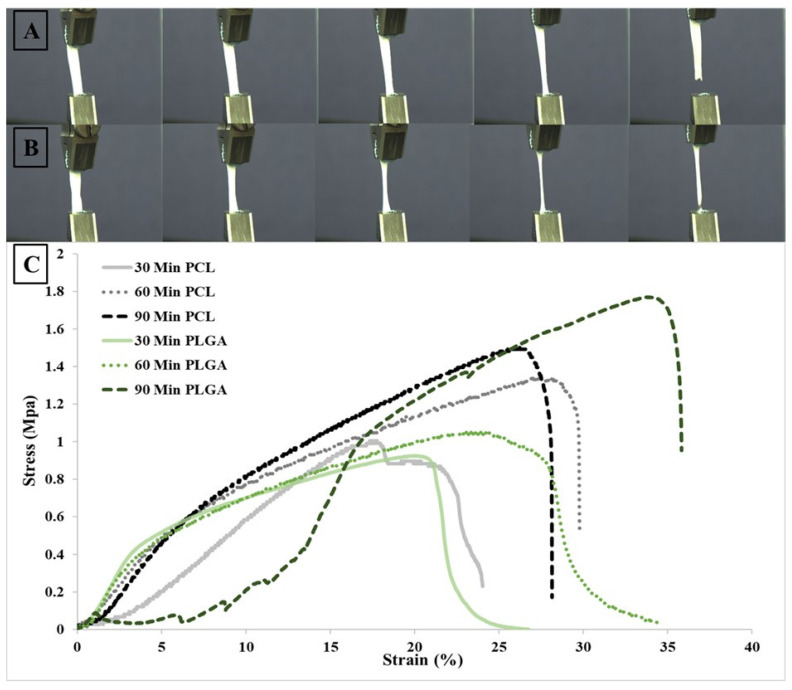
Tensile tests of electrospun PCL and PLGA scaffolds: photographs of flat sheet scaffolds during tensile testing of (**A**) 60-min PCL and (**B**) 60-min PLGA. (**C**) Stress–strain curves of electrospun nanofibrous structures.

Figure 7 illustrates the mechanical behaviour of the electrospun PCL and PLGA samples, and simply by looking at the stress–strain curves, it is easy to conclude that the 90-min PLGA membrane was shown to be stronger and more elastic than the PCL scaffold. However, none of the membranes showed a reliable definite yielding sign, and by comparing the produced strain/stress graph to other tissue-engineered scaffolds, some similarity can be observed [72,73]. The figure below demonstrates a strong correlation between electrospinning processing time and tensile strength; as electrospinning time increased, the outcome was stronger and thicker scaffolds. Regardless of the electrospinning time and type of polymeric scaffold, the elongation values at the break changed significantly with thickness. The average values of the tensile strength, elongation at break and Young’s modulus of the electrospun membranes are reported in Table 4. Considering the 90-min electrospinning time of both PCL and PLGA scaffolds, the PLGA membrane was shown to be more elastic and tougher than the PCL membrane, with 1.76 MPa ± 0.79 SD and 36.33% ± 2.96 SD for the 90-min PLGA scaffold and 1.49 MPa ± 0.37 SD and 28.153% ± 2.94 SD for the PCL scaffold. One of the reasons that might lead porous scaffolds to have greater strength over other porous membranes is reduced pore size, or the gap between interconnected fibres [74,75].

### 3.5. Handleability 

**Figure 8:**
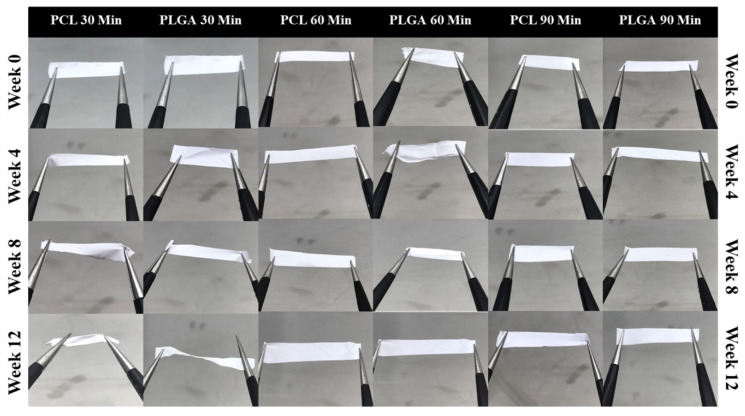
Handleability images of 30-, 60- and 90-min PCL and PLGA scaffolds over a 12-week period.

An ideal scaffold should have many properties including adequate biodegradability and biocompatibility, promotion of cell attachment, and sufficient mechanical properties. However, even if fabricated scaffolds achieve all of the above characteristics, the tissue/organ will not be functional if fabricated tissues were not easy to work with during the surgical procedure. Handleability of the tissue-engineered membrane after the initiation of the degradation process or cell seeding/proliferation in vitro is vitally crucial for bioengineering applications [76]. Figure 8 below shows that the subjective assessment of all membranes’ physical handling after the degradation process showed that membranes had strong physical integrity, indicating that these scaffolds could sustain their structural integrity for long durations.

## 4. Discussion

The fibres produced by the electrospinning process have diameter ranges of 0.1–100 µm, and they can control and change other electrospun membrane structure features [77]. Reducing the fibre diameter will increase the surface porosity, leading to smaller pore areas, and vice versa, affecting specific cells that attach and proliferate [78]. However, in this study, a similar trend was observed during the degradation process: As the fibre diameter increased in size due to water intake, the pore sizes (µm^2^) and the surface area-to-pore ratio decreased substantially. Regardless of the medical application, appropriate degradability is an essential factor that must be considered when designing and manufacturing scaffolds for tissue engineering purposes. As shown in Figure 3, an obvious connection was observed between the electrospinning processing time and mass loss %, and the thicker scaffolds had a more prolonged reduction in their weight loss. For instance, the PCL scaffolds displayed a reduction in weight loss of more than 32% when they were electrospun for an additional 60 min as they increased in thickness from 0.06 mm (30 min) to 0.11 mm (90 min), but the reduction in mass loss (only 6.08%) observed for PLGA scaffolds was not as significant as for the PCL scaffolds. It has also been found that the thickness of the scaffolds also significantly affects the rate of degradation [79].

The collected data also reveal several issues related to degradation patterns, potential phenomena and practicality that must be resolved. The first, and possibly the most critical, issue that needs to be mentioned is the shape of the data itself. The data fluctuated and caused unpleasant errors not only for weight loss measurements, but also when measuring structural morphology, e.g., fibre diameter, pore size and porosity (%). The reason for this apparent variation was mainly related to the characteristics of sample size and weight. The second issue that must be addressed is that the synthetic polymeric scaffolds tended to shrink during the degradation period, and this did not depend on the environmental temperature (either room temperature or in the incubator at 37 °C); for this reason, we can assume that the membrane shrinkage was due to polymer chain relaxation. However, there are several studies that have demonstrated that the scaffolds produced from synthetic biodegradable polymers can have entirely different characteristics than natural polymeric scaffolds [80,81,82,83]. When synthetic polymeric scaffolds are soaked in alkaline solution at a controlled temperature of 37 °C, the fibres tend to swell up in size, which then causes changes in the structural morphology of the scaffolds, as the pore size and pore distribution decreases substantially, which eventually can negatively affect and prevent cell penetration in the pores and proliferate within the extracellular matrix of the scaffolds. Whereas some natural polymer scaffolds show the complete opposite characteristic, as the fibre diameters increase in size, and the pore size/volume ratio increases too, and this feature could be beneficial for colonisation by cells [84,85]. While pore size and pore distribution are crucial for cell migration, proliferation and vascularisation, if these pores are not well connected to each other, it makes them unfunctional due to the vascularisation that might not occur properly [86,87]. 

## 5. Conclusions

To conclude, this study’s results indicated that crosslinking was successfully achieved and observed in all electrospun mats and had a positive impact on the mechanical properties of the scaffolds and increased their integrity over the degradation period. Due to the scaffolds’ size, weight and thickness, their mechanical properties were adequate for the tissue engineering process. However, both polymer scaffolds’ characteristics in this study can be adjusted by changing some manufacturing methods through the electrospinning process. Both types of scaffolds displayed more robust mechanical properties when increasing the spinning times. The tensile strength of both scaffolds with 90 min electrospun membranes did not show a significant difference, as the PCL and PLGA scaffolds measured at 1.492 MPa ± 0.378 SD and 1.764 MPa ± 0.7982 SD, respectively. All membranes were shown to be hydrophobic under the wettability test. Further scaffold optimisation is needed to either increase scaffold hydrophilicity or increase the crystallinity of both PCL and PLGA membranes to prevent shrinkage in PBS solution or as needed in further studies such as for in vitro cell works. Performing optimisation should be done with extra care to make sure not to reduce the fabricated membranes’ mechanical integrity.

## Figures and Tables

**Table 1:** Electrospinning process parameters that were recorded during the experiment.

Electrospinning	Sample Name	Voltage (kV)	Needle Type	Distance from Tip of the Needle to the Collector (mm)	Type of Collector	Flow Rate (mL/h)	T (°C)	Humidity (%)	Time (min)	Solution Dispensed (mL)
PCL Only	A1	7.90	20 G	95	Flat	1	22.1	46	30	0.498
	A2	7.91	20 G	95	Flat	1	22.3	46	60	1.01
	A3	7.36	20 G	95	Flat	1	22.3	44	90	1.507
PLGA Only	B1	7.90	20 G	95	Flat	1	22.2	46	30	0.509
	B2	8.56	20 G	95	Flat	1	23.1	39	60	1.005
	B3	8.74	20 G	95	Flat	1	23.1	39	90	1.576

**Table 2:** Mean ± SEM of contact angle measurements of PCL, PLGA scaffolds.

Scheme		Mean ± SD (DH2O)	
		Left Angle	Right Angle
PCL	30 min (A1)	122.77° ± 3.72	122.40° ± 4.51
	60 min (A2)	111.40° ± 3.23	123.13° ± 2.70
	90 min (A3)	125.57° ± 4.75	125.07° ± 4.80
PLGA	30 min (B1)	127.83° ± 6.16	129.03° ± 3.55
	60 min (B2)	131.73° ± 3.46	132.73° ± 3.32
	90 min (B3)	129.83° ± 5.93	129.27° ± 5.60

**Table 3:** (A) Percentage change in fibre dimeter of PCL and PLGA scaffolds at week 12 of degradation compared to week 0, (B) Percentage change in pore size for PCL and PLGA scaffolds electrospun for 30, 60, and 90 minutes, (C) Percentage change in surface porosity percentage for PCL and PLGA scaffolds electrospun for 30, 60, and 90 minutes. (−) = decrease and (+) = increase.**

A	Percentage Change in Fibre Diameter		
	30 Min	60 min	90 min
PCL 12 Weeks	9.04 (−)	34.50 (+)	1.72 (−)
PLGA 12 Weeks	35.60 (+)	25.07 (+)	1.38 (−)
PCL 4 Weeks at 37 ^o^C	33.60 (+)	14.09 (+)	28.13 (+)
PLGA 4 Weeks at 37 ^o^C	88.07 (+)	32.95 (+)	50.48 (+)
B	Percentage Change in Pore size		
	30 Min	60 min	90 min
PCL 12 Weeks	88.03 (−)	83.86 (−)	82.85 (−)
PLGA 12 Weeks	86.61 (−)	81.09 (−)	84.97 (−)
PCL 4 Weeks at 37 ^o^C	54.52 (−)	35.39 (−)	54.21 (−)
PLGA 4 Weeks at 37 ^o^C	39.54 (−)	46.76 (−)	60.18 (−)
C	Percentage Change in Surface Porosity (%)		
	30 Min	60 min	90 min
PCL 12 Weeks	88.21 (−)	87.97 (−)	81.57 (−)
PLGA 12 Weeks	86.74 (−)	83.64 (−)	85.79 (−)
PCL 4 Weeks at 37 ^o^C	33.25 (−)	23.55 (−)	36.95 (−)
PLGA 4 Weeks at 37 ^o^C	53.64 (−)	40.41 (−)	41.762 (−)

**Table 4:** Summary of mechanical properties for PCL and PLGA solutions electrospun for 30, 60 and 90 min.

Sample Name	Time	Length (mm)	Thickness (mm)	Width (mm)	Area (mm^2^)	Tensile Strength (MPa ± SD)	Elongation at Break (% ± SD)	Young Modulus (MPa ± SD)
PCL	30	35	0.06	6	0.36	0.99 ± 0.17	24.03 ± 2.24	8.07 ± 2.14
	60	37.31	0.09	5.6	0.504	1.32 ± 0.49	29.83 ± 3.9	11.71 ± 2.96
	90	37.2	0.11	5.69	0.6259	1.49 ± 0.37	28.15 ± 2.94	13.69 ± 3.14
PLGA	30	35	0.09	5.8	0.522	1.03 ± 0.25	34.36 ± 5.77	10.15 ± 1.64
	60	35	0.12	6	0.72	0.92 ± 0.45	21.74 ± 3.28	9.64 ± 2.17
	90	35	0.12	5.9	0.708	1.76 ± 0.79	36.33 ± 2.96	15.15 ± 5.14

## Data Availability

Not applicable.
